# Association of Interleukin 7 Immunotherapy With Lymphocyte Counts Among Patients With Severe Coronavirus Disease 2019 (COVID-19)

**DOI:** 10.1001/jamanetworkopen.2020.16485

**Published:** 2020-07-22

**Authors:** Pierre Francois Laterre, Bruno François, Christine Collienne, Philippe Hantson, Robin Jeannet, Kenneth E. Remy, Richard S. Hotchkiss

**Affiliations:** 1 Department of Critical Care Medicine, Saint Luc University Hospital, Université Catholique de Louvain, Brussels, Belgium; 2 Intensive Care Unit Department and Inserm Centre Investigation Clinique 1435 and Unité Mixte de Recherche 1092, University Hospital of Limoges, Limoges, France; 3 Department of Intensive Care Medicine and Anaesthesiology, University Hospital of Limoges, Limoges, France; 4 Department of Pediatrics, Washington University School of Medicine in St Louis, St Louis, Missouri; 5 Department of Anesthesiology and Critical Care Medicine, Washington University School of Medicine in St Louis, St Louis, Missouri; 6 Department of Medicine, Washington University School of Medicine in St Louis, St Louis, Missouri; 7 Department of Surgery, Washington University School of Medicine in St Louis, St Louis, Missouri

## Abstract

This case series examines whether interleukin 7 (IL-7) is associated with restored host protective immunity in patients with severe coronavirus disease 2019 (COVID-19) and immunosuppression.

## Introduction

Cytokine storm–mediated organ injury continues to dominate current thinking as the primary mechanism for coronavirus disease 2019 (COVID-19). Although there is an initial hyper-inflammatory phase, mounting evidence suggests that virus-induced defective host immunity may be the real cause of death in many patients.^1,2^

COVID-19 has been called a serial lymphocyte killer because profound and protracted lymphopenia is a near uniform finding among patients with severe COVID-19 and correlates with morbidity and mortality.^1,3^ Autopsies demonstrate a devastating depletion of lymphocytes in the spleen and other organs.^2^ CD4, CD8, and natural killer cells, which play important antiviral roles, are depleted and have reduced function, leading to immune collapse.^1^

Clinical and pathological findings in patients with COVID-19 indicate that immunosuppression is a critical determinant of outcomes. Secondary hospital-acquired infections occur in 50% of patients who die.^1^ Cell inclusion bodies, consistent with viral persistence, have been found in lungs, kidneys, and other organs.^1,2^ Severe acute respiratory syndrome coronavirus 2 (SARS-CoV-2)–specific quantitative polymerase chain reaction has revealed viral load in the lungs of most patients at autopsy, consistent with an inability to eliminate the pathogen.^1,2^ Collectively, these studies indicate that impaired immune competence is an important pathogenic mechanism in COVID-19.

Interleukin 7 (IL-7) is a pleiotropic cytokine essential for lymphocyte survival and expansion.^4,5^ Administration of IL-7 invariably increases circulating and tissue lymphocytes and has been administered to more than 450 patients with an excellent safety profile.^4,5^ IL-7 is currently in multiple randomized clinical trials for oncologic and infectious disorders, and a trial in the United Kingdom is evaluating its use among patients with severe COVID-19. Importantly, IL-7 has documented efficacy as an antiviral agent.^4,5^ IL-7 therapy has been shown to restore lymphocyte counts and functional activity, leading to decreased viral load and clinical improvement in several life-threating viral infections.^4,5^ It has been shown to increase CD4 and CD8 T-cells 3-fold, to improve T-cell activation, to not increase proinflammatory cytokines, and to be well tolerated in patients with bacterial sepsis.^6^ Thus, a compelling scientific rationale exists for examining whether IL-7 is associated with restored host protective immunity in patients with COVID-19 and immunosuppression and improve outcomes.^1^

## Methods

In this case series, IL-7 was approved by the Ethics Committee of St Luc University Hospital (Brussels, Belgium) for compassionate use in 12 critically ill patients with COVID-19 and severe lymphopenia who had 2 consecutive absolute lymphocyte counts of less than 700/μL (to convert to ×10^9^ per microliter, mutliply by 0.001). Patients provided written informed consent. This study followed the Appropriate Use and Reporting of Uncontrolled Case Series in the Medical Literature reporting guideline for case series. An initial safety dose of 3 μg/kg was followed by a dose of 10 μg/kg by intramuscular injection twice a week for 2 weeks. A total of 13 patients with COVID-19 who received standard-of-care treatment and were matched to severity of illness, comorbidities, and other factors were included as a comparator control cohort to the IL-7 treatment group.

Data were analyzed using a mixed-effects model with repeated measures and the autoregression 1 covariance structure. The logs of the absolute lymphocyte scores were used because they provided a better fit to the model. The associations of group, study day, and the group by study day interaction were all significant. Statistical analyses were conducted in SAS version 9.4 (SAS Institute). Statistical significance was set at *P* < .05, and all tests were 2-tailed.

## Results

The 12 patients in the IL-7 group had a mean (range) age of 62 (48-76) years, and 11 (92%) were men. The 13 patients in the control group had a mean (range) age of 59 (42-83) years, and 9 (69%) were men (Table). No observed treatment-associated adverse effects were noted, and IL-7 was well tolerated without inducing changes in temperature, blood pressure, or Pao_2_:Fio_2_ ratio (Figure). Plasma proinflammatory cytokines were measured before and at 7 and 24 hours after IL-7 administration to determine whether IL-7 induced hyper-cytokinemia. Administration of IL-7 was not associated with changes in tumor necrosis factor α, IL-1β, and IL-12p70 concentrations, which were below the level of detection at all time points in all patients. There was no consistent association of IL-7 administration with IL-6 concentration. Overall, 3 patients (25%) had decreases in IL-6 concentrations and 2 patients (17% ) had increases in IL-6 concentration of approximately 1000 to 1500 pg/mL at 7 hours after administration of IL-7, suggesting that these changes in IL-6 concentration may have been because of changes in the underlying patient physiologic state rather than because of IL-7. At day 30, secondary infections occurred in 7 patients (58%) in the IL-7 group compared with 11 (85%) in the control group; 30-day mortality was 42% (5) and 46% (6), respectively (Table). IL-7 was associated with a restored lymphocyte count, with the IL-7 group having levels more than 2-fold greater than the control group (mean [SE], 1734/μL [227/μL] vs 885/μL [239/μL]; *P* = .02) (Figure).

**Table.** Patient Characteristics

Characteristic	No. (%)	
	IL-7 group (n = 12)	Control group (n = 13)
Age, mean (range), y	62 (48-76)	59 (42-83)
Sex		
Women	1 (8)	4 (31)
Men	11 (92)	9 (69)
BMI, mean (range)	25.13 (0-33.95)	27.33 (21.42-37.37)
Comorbidities		
Hypertension	6 (50)	6 (46)
Diabetes	2 (17)	2 (15)
Cardiopathy	2 (17)	2 (15)
Other	6 (50)	8 (62)
APACHE II score, mean (range)	14 (9-20)	13 (7-16)
SOFA score, mean (range)	5 (4-7)	4 (2-5)
Worst Pao_2_:Fio_2_ ratio, mean (range)	89 (49-182)	83 (54-180)
CRP at admission, mean (range), mg/dL	20.1 (0.74-42.3)	1.98 (0.33-48.0)
Time from ICU admission to intubation, mean (range), d	3 (0-8)	3 (0-9)^a^
Secondary infection(s)	7 (58)	11 (85)
30-day mortality	5 (42)	6 (46)

Abbreviations: APACHE, Acute Physiology and Chronic Health Evaluation; BMI, body mass index (calculated as weight in kilograms divided by height in meters squared); CRP, C-reactive protein; ICU, intensive care unit; SOFA, Sequential Organ Failure Assessment.

SI conversion factor: To convert CRP to mg/L, multiply by 10.

^a^ 1 of 13 patients was never intubated.

**Figure.**
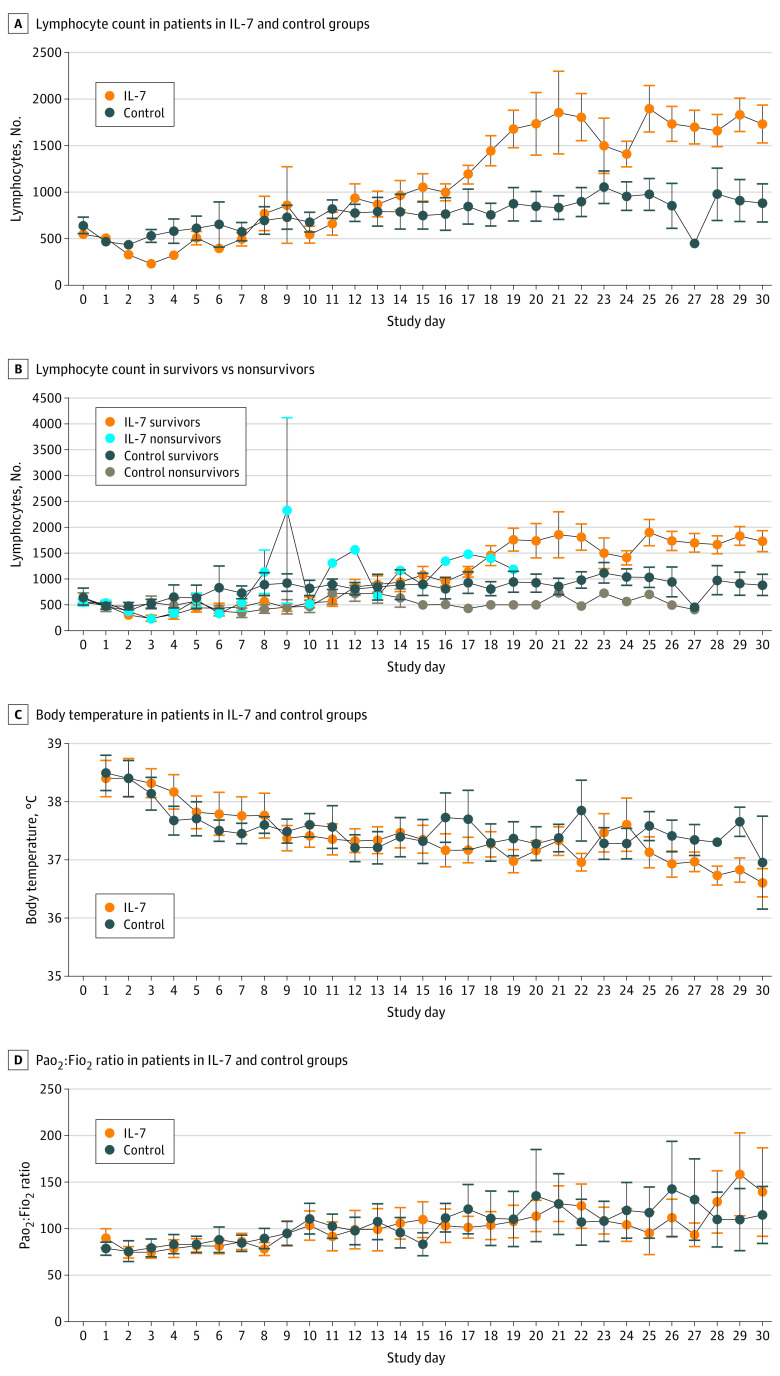
Association of Interleukin 7 (IL-7) With Absolute Lymphocyte Count, Body Temperature, and Pao_2_:Fio_2_ Ratio IL-7 was administered at 3 μg/kg on day 1 and at 10 μg/kg at days 2, 5, 8, and 12. Points indicate means, and whiskers indicate SEs.

## Discussion

The findings of this study suggest that IL-7 can be safely administered to critically ill patients with COVID-19 without exacerbating inflammation or pulmonary injury. IL-7 was associated with lymphocytes returning to a reference level, appearing to reverse a pathologic hallmark of COVID-19. Importantly, previous studies have reported that IL-7–associated restoration of lymphocyte numbers enhanced the activity of antiviral agents.^6^ A limitation of the current study is that no phenotypic or functional studies of immune cells were conducted. Administration of IL-7 alone or in combination with other therapies warrants serious consideration for patients with COVID-19 and evidence of immunosuppression.
